# Hemodynamic Data Assimilation in a Subject-specific Circle of Willis Geometry

**DOI:** 10.1007/s00062-020-00959-2

**Published:** 2020-09-24

**Authors:** Franziska Gaidzik, Sahani Pathiraja, Sylvia Saalfeld, Daniel Stucht, Oliver Speck, Dominique Thévenin, Gábor Janiga

**Affiliations:** 1 Lab. of Fluid Dynamics and Technical Flows, Otto von Guericke University Magdeburg, Magdeburg, Germany; 2 Institute for Mathematics, University of Potsdam, Potsdam, Germany; 3 Department of Simulation and Graphics, Otto von Guericke University Magdeburg, Magdeburg, Germany; 4 Institute for Physics, Otto von Guericke University Magdeburg, Magdeburg, Germany; 5 Institute of Biometry and Medical Informatics, Otto von Guericke University Magdeburg, Magdeburg, Germany; 6 Leibniz Institute for Neurobiology, Magdeburg, Germany

**Keywords:** Hemodynamics, CFD, Uncertainty Quantification, PC-MRI, LETKF

## Abstract

**Purpose:**

The anatomy of the circle of Willis (CoW), the brain’s main arterial blood supply system, strongly differs between individuals, resulting in highly variable flow fields and intracranial vascularization patterns. To predict subject-specific hemodynamics with high certainty, we propose a data assimilation (DA) approach that merges fully 4D phase-contrast magnetic resonance imaging (PC-MRI) data with a numerical model in the form of computational fluid dynamics (CFD) simulations.

**Methods:**

To the best of our knowledge, this study is the first to provide a transient state estimate for the three-dimensional velocity field in a subject-specific CoW geometry using DA. High-resolution velocity state estimates are obtained using the local ensemble transform Kalman filter (LETKF).

**Results:**

Quantitative evaluation shows a considerable reduction (up to 90%) in the uncertainty of the velocity field state estimate after the data assimilation step. Velocity values in vessel areas that are below the resolution of the PC-MRI data (e.g., in posterior communicating arteries) are provided. Furthermore, the uncertainty of the analysis-based wall shear stress distribution is reduced by a factor of 2 for the data assimilation approach when compared to the CFD model alone.

**Conclusion:**

This study demonstrates the potential of data assimilation to provide detailed information on vascular flow, and to reduce the uncertainty in such estimates by combining various sources of data in a statistically appropriate fashion.

## Introduction

Recent studies have investigated the influence of hippocampal vascularization patterns on cognitive performance [1], yet very little is known about the relationship between the interaction of cerebral blood flow and vascular profiles on cognition. The circle of Willis (CoW) is the primary collateral pathway for cerebral vasculature [2] and its hemodynamics provide a representation of the global intracranial vascularization structure. Its anatomy varies significantly between individual subjects. Often there is a lack of communication between the four input vessels (internal carotid and vertebral arteries), due to occlusion in one of the vessel branches or even a lack of the branch as a normal variant [3]. This high variability necessitates subject-specific investigation of cerebral blood flow, which is difficult to assess and often the resulting flow estimates show considerable uncertainties. The flow state in intracranial arteries can be estimated using various methods. Phase-contrast magnetic resonance imaging (PC-MRI) measurements can be used to obtain time-dependent quantitative velocity fields but are impaired by low signal-to-noise ratios (SNR), as well as limited spatial and temporal resolution [4, 5]. An alternative to in vivo measurements is the use of computational fluid dynamics simulations (CFD) to extract subject-specific flow information [3, 6, 7]. Such an approach is advantageous since the resulting state estimate satisfies conservation laws and captures even fine intracranial arteries, but it is highly dependent on the specification of correct initial and boundary conditions, such as the exact vessel geometry and flow input [8, 9]. Contrary to flow investigation in aneurysms, existing flow studies in complex vascular pathways provide only simple comparisons between measurements and simulations, or use either of the two alone for flow quantification [10–12]. Inflow and outflow conditions are one of the major unknowns when constructing the numerical model [13]; often the associated uncertainty and the corresponding impact on flow estimates is not quantified [14]. Approaches that are more sophisticated use available PC-MRI data as initial conditions for CFD simulations [3, 6]; however, no study incorporates the entire 3D velocity field into the numerical model.

In this study, we demonstrate the potential for data assimilation (DA) to provide improved state estimates by merging measurement data with numerical model simulations based on their respective uncertainties. In the setting of Gaussian distributed variables, DA produces state estimates with lower uncertainty (variance) than both the measurement data and model simulations. Additionally, the temporal and spatial resolution of measured parameters can be increased. Although widely used in many areas of science and engineering, the application of DA in hemodynamics is still in its infancy. Only a handful of studies have considered intracranial aneurysms, using either sequential or variational DA approaches [15–21]. Variational methods can be computationally expensive due to the adjoint equations, which are about twice as costly to solve as the direct equations, and often require several iterations until convergence. Consequently, most DA studies consider only steady-state flow conditions and/or 2D geometries. The few transient approaches for DA in aneurysms are limited by the low spatial resolution of the numerical model [19, 20]. The computational cost of DA becomes even more of an issue for highly complex geometries, such as the CoW, since the extent of vessel branches that are included in the model greatly influences runtime [22]. In this study, we use the local ensemble transform Kalman filter (LETKF) [23] to improve hemodynamic flow estimates in the CoW, compared to either measurement data or CFD simulations alone. The LETKF, a method in sequential DA, is well-suited to this application, since it can handle the non-linearity of the Navier-Stokes equations and samples the system state and covariance matrices by an ensemble of CFD simulations. Similar to the original ensemble Kalman filter [24], the LETKF directly provides an estimate of the system uncertainty by the spread of the ensemble members. The localization procedure inherent in the LETKF enables parallel computation and reduces the number of ensemble members needed for a statistical representation of the state estimate.

To the best of our knowledge, this study is the first to provide a transient state estimate for the 3D velocity field in a subject-specific CoW geometry using DA. By including the DA step, we gain detailed information about the intracranial vascular supply that comes together with an uncertainty quantification of related parameters. This study demonstrates DA for a subject-specific intracranial model with high complexity (i.e., small vessel radii, multiple inlets and outlets) and makes use of all obtained 4D PC-MRI flow data to generate an improved state estimate.

## Modeling and Data Assimilation

### Measurement Data

The complete anatomy of the classical CoW, including all connecting arteries, is presented in this study. The measurement data, in the form of PC-MRI flow fields, is used for the segmentation of the modeling geometry, comparison of state estimates, and most importantly, serves as observations ($y_{\mathrm{obs}}$) in the DA experiment. The 4D flow data were acquired on a healthy volunteer using a 7 T whole-body MRI system (Siemens Healthineers, Erlangen, Germany) in a 32-channel head coil (Nova Medical, Wilmington, MA, USA) using 4D PC-MRI. The acquisition sequence was based on an radio-frequency(RF)-spoiled gradient echo with quantitative flow encoding in all three spatial dimensions [5, 25]. Information on electrocardiogram (ECG) gating was delivered by an acoustic cardiac gating device (MRI.Tools GmbH, Berlin, Germany). For each of the resulting 17 time steps, 3 velocity maps are available that contain information on the *x*-, *y*-, and *z*-velocity components, as well as one structural magnitude image. The acquired image data represent an isotropic voxel size of 0.64 mm and a temporal resolution of 54.4 ms. The velocity encoding parameter (VENC) that defines the highest velocity that is encoded uniquely in the phase was set to 0.9 m/s. The SNR was found to be approximately 55. Postprocessing of the acquired raw data was undertaken using MeVisLab 2.3.1 (MeVis, Bremen, Germany) and the automated tool described in Bock et al. [26]. This includes noise masking, antialiasing and the conversion to the EnSight (ANSYS Inc., Canonsburg, PA, USA) file format. Phase-wraps that occurred during peak systolic flows in both middle cerebral arteries have been manually corrected within the postprocessing pipeline. In addition to the PC-MRI acquisition, high-resolution (0.32 mm isotropic) time-of-flight (ToF) imaging was performed on the same healthy volunteer. The ToF technique is particularly suited to image vascular structures. A prospective motion correction system was applied for both the 4D PC-MRI scan and the ToF scan [27–31]. As the correction was only applied in-scan, not inter-scan, registration of both scans was still necessary.

### Numerical Model

The blood flow is fully governed by the non-linear Navier-Stokes equations. They are solved using the finite volume solver STAR-CCM +14.04 (Siemens Product Lifecycle Management Software Inc., Plano, TX, USA). The numerical mesh consisted of ~4.3 million polyhedral and prismatic cells, having a base size of 0.1 mm and 5 prism layers at the wall. The vessel walls are assumed to be rigid and blood is considered as an incompressible (ρ = 1055 kg/m^3^), Newtonian (η = 4 mPas) fluid. The time-step for the underlying laminar, transient simulations is chosen to be t = 1 ms. Specification of inflow and outflow conditions, and their uncertainty is discussed in Sect. “Uncertainty Quantification”. The segmentation of the model geometry is described below.

#### Geometry Segmentation

**Fig. 1:**
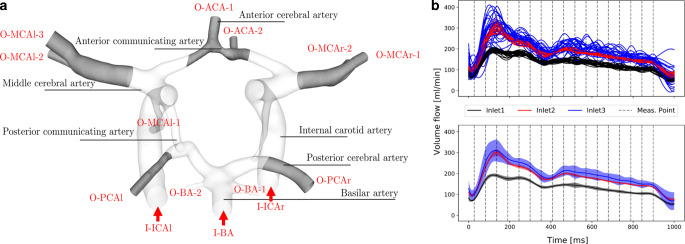
**a** Overview of the investigated circle of Willis of a healthy volunteer. Main supplying vessels are labeled and inlets (I) and outlets (O) are specified. The grey shaded areas are used for the comparison of the wall shear stress distribution in Fig. 3b. **b** Sampled inflow trajectories for the ensemble CFD simulations. *Top*: overlaid inflow trajectories for the simulated ensemble members, *bottom*: variance of the boundary conditions displayed as shaded areas around the corresponding mean value

The 3D surface segmentation was extracted from the 4D PC-MRI data. We propose the following segmentation procedure, which was used to enhance the SNR of the data. First, the phase as well as the magnitude images were temporally averaged, yielding only a single 3D volume each. Next, the phase images were combined and multiplied with the temporally averaged magnitude image. Then, thresholding was applied in order to extract the vessel surface. For surface extraction, the marching cubes algorithm was applied within the MeVisLab framework [32]. In order to remove artifacts on the resulting surface mesh, corrections on subvoxel level were executed, as described in [33]. For validation of the segmented 3D surface model, we also carried out a segmentation of the ToF 7 T MRI dataset by applying the Frangi filter [34]. Although we observed very good agreement in the larger arteries, displacements between the surface meshes from the ToF and the PC data were present in the peripheral arteries. Therefore, we chose the PC-MRI surface. For the posterior communicating arteries (characterized by smaller diameters compared to internal carotid, anterior and middle cerebral arteries), the correction of the surface mesh was guided by the ToF segmentation. Finally, the inlets and outlets of the 3D vessel model were cut perpendicular to the vessel center line and extruded to reduce the influence of boundary conditions. An overview of the segmented model geometry, together with the nomenclature of the inlet and outlet parts is given in Fig. 1a.

### Data Assimilation Algorithm

The standard DA problem consists of two components. Firstly, a dynamic system related to a state vector $x_{t}$ and a model for its time evolution (e.g., Navier-Stokes equations with velocity state variables), and secondly noisy observations of the system states (e.g., PC-MRI velocity data) according to

1 $$y_{t}^{\mathrm{obs}}=h\left(x_{t}\right)+\epsilon _{t}$$

where $\epsilon _{t}$ is a noise term and $h$ the observation operator that relates the system states $x_{t}$ to the observation variables. The goal of DA is to combine model simulations of $x_{t}$ (referred to as the background) and observations $y_{t}^{\mathrm{obs}}$ to obtain an improved estimate of the system states (referred to as the analysis) at any given time $t$. The Kalman filter [35] provides the optimal state estimate for linear systems and Gaussian errors. Its Monte Carlo extension to non-linear systems, the EnKF [24] has become widely popular as a relatively accurate and robust DA method for high dimensional applications. The LETKF [23] is an ensemble Kalman type algorithm belonging to the class of deterministic square root filters. Hence, the square root of the analysis error covariance matrix is used to produce the analysis in such a way that the analysis covariance matches that of the Kalman filter. It also has the advantage of avoiding the direct calculation of the high dimensional analysis sample covariance matrix, as the computations are transformed into ensemble space, which is of lower dimensionality. The square root filter equations are as follows:

2 $$\overline{x}_{t}^{a}=\overline{x}_{t}^{b}+K_{t}\left(y_{t}^{\mathrm{obs}}-h\left(\overline{x}_{t}^{b}\right)\right)$$

3 $$X_{t}^{a}=X_{t}^{b}W_{t}^{a}$$

4 $$\tilde{P}_{t}^{a}=\left[\left(k-1\right)I+\left(Y_{t}^{b}\right)^{T}R_{t}^{-1}Y_{t}^{b}\right]^{-1}$$

5 $$W_{t}^{a}{W_{t}^{a}}^{T}=(N-1)\tilde{P}_{t}^{a}$$

6 $$x_{t}^{a,i}=\overline{x}_{t}^{a}+X_{t}^{a,i}$$

where $\overline{x}_{t}^{b}$ is the background ensemble mean, i.e., the sample mean of model simulations of the state $x_{t}$ at time $t$; $\overline{x}_{t}^{a}$ is the analysis ensemble mean, i.e., the sample mean of the state after combining simulations and measurements; $X_{t}$ is the matrix of ensemble perturbations from the mean and $Y_{t}^{b}$ the matrix of ensemble perturbation in observation space; $\tilde{P}_{t}^{a}$ is the analysis covariance matrix in ensemble space; $N$ is the number of ensembles; $K_{t}$ is the Kalman gain matrix, which provides the appropriate weighting of observations and model simulations based on their uncertainties; $W_{t}^{a}$ is determined using the eigenvectors and eigenvalues of $\tilde{P}_{t}^{a}$ and refers to the perturbations in the ensemble space and $x_{t}^{a,i}$ indicates the $i$-th ensemble member. The analysis mean is reconstructed The LETKF calculates local analyses by taking only observations in a predefined neighborhood of each state grid point into account. Thus, Eqs. 2, 3 4 and 5 are transformed to a local scale and are repeated for each model grid point [23]. This enables efficient parallel computation and avoids spurious correlations between distant observations.

The original algorithm has been adapted to match the requirements of this hemodynamic application. In a previous study, the LETKF has been validated against highly resolved ground truth measurement data in a synthetic aneurysm model [20]. Although the CoW geometry is more complex, the results of the ideal aneurysm case provide guidelines for the DA settings (e.g., ensemble size, observation operator, localization radius) in this study. The observation operator was defined to be a spatial binning operator that maps the model variables to the lower resolution observation space. A sensitivity analysis showed that a localization radius of 7 mm produced reliable local analyses. We have chosen an ensemble size of 25 to balance computational expense and accuracy. The data assimilation time window was given by the temporal resolution of the measurement data; observations are available every 54.4 ms.

#### Uncertainty Quantification

A successful implementation of the DA step strongly depends on the correct description of background and observation uncertainty. We used the velocity variance of the PC-MRI measurements as an indication of the observation uncertainty. Based on Pelc et al. [4], the velocity variance is directly related to the VENC and the SNR in corresponding magnitude images, $\sigma _{v}=\frac{\sqrt{2}}{\pi }\frac{VENC}{SNR}$. Uncertainty in the numerical simulations arises from initial and boundary conditions, captured by the inflow and outflow ensemble. Flows are assumed to be distributed according to independent Gaussian curves, with mean and standard deviation approximated by the sample statistics of flow rates extracted from the original PC-MRI data at 10 different positions in each inlet or outlet vessel at each time point. Ensembles were generated at each time step by sampling from these Gaussians, with cubic interpolation used to generate a continuous trajectory (Fig. 1b). A flow-split outlet boundary condition was used in the ensemble CFD simulations. Specifically, outflows were sampled consistently with the inflow rates, and transformed to ratios by dividing by the sum of outflows over all outlets for each sample.

## Results

### Qualitative Results

**Fig. 2:**
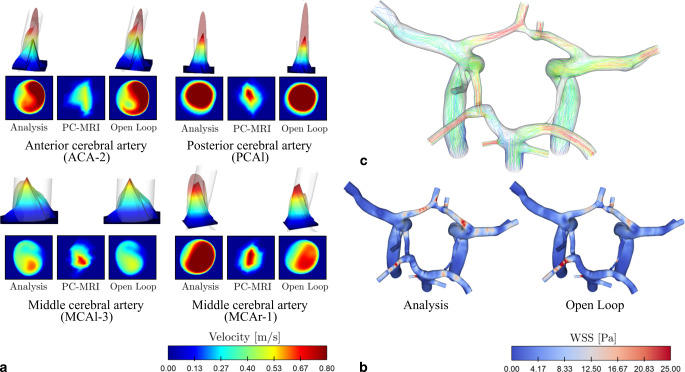
Qualitative comparison of peak-systolic flow. **a** Velocity profiles at characteristic locations in the vascular system are compared between analysis, open loop and PC-MRI. Numerically obtained flow profiles (analysis, open loop) use transparent visualizations; the PC-MRI flow data is displayed opaquely. The bottom row corresponds to the cross-section of the velocity distribution in the respective vessel and the top row corresponds to the elevated surface of this distribution. **b** Wall shear stress (WSS) distribution over the complete CoW for both analysis and open loop **c** Analysis-based streamlines plotted in the complete circulatory system

The peak systolic flow is compared between PC-MRI data, open loop calculation and analysis estimate. The open loop refers to the state estimate that is obtained by an average of the purely numerical background simulation with no DA. Fig. 2a displays the velocity profiles at four characteristic locations (see Fig. 1a) in the circulatory flow system. Numerical flow profiles (analysis and open loop) are displayed semi-transparently and are overlaid with an opaque representation of the PC-MRI measurements. The corresponding cross-sectional velocity magnitudes are displayed below. In general, the data assimilation step shifts the estimated flow profiles closer to the measurements but keeps the shape similar to the open loop. Because of their higher resolution, analysis and open loop flow profiles appear to be smoother in comparison to the measurement-based profiles. This effect is most pronounced for velocity values near the vessel walls, leading to a better representation of geometry edges.

The resolution of PC-MRI is not sufficient for the calculation of wall shear stress (WSS) on the CoW wall surface using direct approaches based on the velocity gradient at the wall surface; however, WSS can be derived using the analysis state estimates, which are available at CFD grid resolution, using the stream-wise component of the velocity gradient in the wall-normal direction. The qualitative comparison between both distributions (Fig. 2b) shows an increase in WSS for the analysis calculation in the anterior part of the CoW, as well as in the middle cerebral arteries. Left and right posterior arteries have higher WSS values for the open loop estimate. These findings are consistent with the qualitative evaluation of the velocity distribution.

The streamlines displayed in Fig. 2c give a general overview of the flow behavior within the CoW. They are calculated based on the analysis flow field and show smooth 3D flow trajectories. It is important to note that both posterior communicating arteries connect the anterior and posterior part of the CoW, but the flow can only be observed in the left part.

### Quantitative Results

**Fig. 3:**
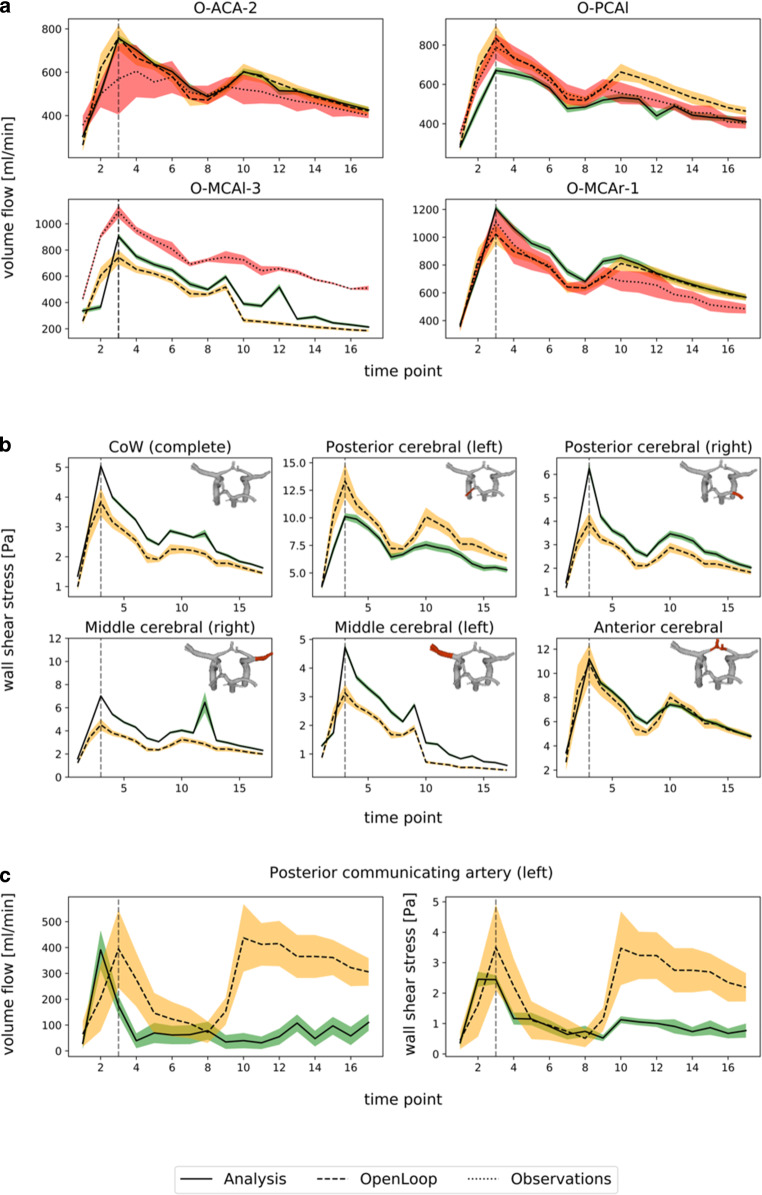
Quantitative comparison between the different modalities for quantities of interest. The peak-systolic flow is illustrated with a vertical grey line. **a** The estimate and spread of volume flow in four different arteries is compared between analysis, open loop and observations. The band around the lines corresponds to the calculated variance of the state estimate (*red*: observations; *orange*: open loop; *green*: analysis). **b** The averaged WSS in six different regions of the underlying geometry is compared between open loop and analysis. **c** Flow rate and WSS in the left posterior communicating artery

The analysis ensemble mean $\overline{x}_{t}^{a}$ provides an estimate of the true vascular velocity field, and the ensemble spread gives an estimate of the uncertainty. Fig. 3a provides an overview of the outflow estimates of all measurement time points from the analysis, open loop and measurements. Both middle cerebral arteries, the left posterior cerebral artery and one anterior cerebral artery have been chosen for comparison. In most cases, the ensemble mean is in the range of the observations and the open loop, except for the left middle cerebral artery (O-MCAl-3). For all four outflows considered (O-ACA‑2, O‑PCAl, O‑MCAl‑3, O‑MCAr-1), a significant reduction in the variance of the state estimate after the DA step is observed. The highest reduction is found to be in the anterior communication artery. There, the analysis state estimate reduces the uncertainty at peak systolic flow by 90% in comparison to the observations, and by 73% in comparison to the open loop simulation. The lowest reduction in uncertainty is observed in the left middle cerebral artery, and still corresponds to a reduction by 56% compared to observations and by 60% compared to open loop. Fig. 3b displays the average WSS for different parts of the CoW. On average, the uncertainty concerning WSS is reduced by a factor of 2 after DA. Overall, the averaged WSS over the whole CoW is slightly higher (5–30%) for the analysis compared to the open loop prediction, except for the anterior part where both show similar distributions. Using DA, Fig. 3c reveals a significantly lower volume flow through the left posterior communicating artery compared to the open loop prediction, which is qualitatively more consistent with the low signal intensities measured there during PC-MRI acquisition.

## Discussion

The DA experiment has shown to be beneficial in a number of ways. The qualitative results (Fig. 2) give an indication of the vascular communication in the underlying geometry. As it is depicted in the diagrams in Fig. 3, DA leads to a strong reduction in uncertainty concerning flow rates and wall shear stress. Second, the limited image resolution of PC-MRI leads to areas in which no information from the measurements can be gained at all (e.g., posterior communicating arteries). The open loop can already resolve the very small arteries of the CoW, but with a large uncertainty. By combining the two sources of data (measurements and model), we obtain velocity values for the entire geometry while keeping the uncertainty low. The quantitative evaluation of the outflow rate was focused on the supplying arteries for smaller intracranial regions, e.g., the hippocampal vascular supply. The analysis velocities here can be used as improved initial conditions for further modeling in smaller arteries. This can help to gain reliable information about the intracranial hemodynamics and to connect that information to diseases related to the hippocampal vascular supply. Furthermore, quantitative hemodynamic parameter evaluation, e.g., assessment of WSS or inflow jets, play a crucial role for computer-based evaluation of cerebrovascular diseases, e.g., intracranial aneurysms. An important parameter for the assessment of rupture risk is the distribution of wall shear stress. The DA enables the estimation of this important quantity with strongly reduced uncertainty in comparison to a purely numerical model run, which would be beneficial for subject-specific aneurysm research. Finally, computational times are not significantly longer for the data assimilation compared to the open-loop simulation. The analysis calculation for each of the observation time points requires approximately 17 × 4 CPU hours, on top of the transient CFD simulation for all ensemble members (25 × 20 CPU hours). The easy to parallelize nature of the LETKF, as well as the possibility to use the numerical model in a black box that enables user-defined numerical models are the main advantages of the LETKF in comparison to optimization-based approaches such as variational DA. Nevertheless, the computational time needs to be further optimized to ensure applicability of the data assimilation approach in a clinical setting (e.g., using surrogate models instead of high-resolution CFD simulations).

This study is a first step for assessing the benefits of DA for complex intracranial vessel geometries, although some limitations should be noted. First, a ground truth (i.e., a very accurate estimate of the reality, for example from a more sophisticated measuring device) is unavailable, which complicates the verification of the analysis; however, the results have clearly shown a reduction in uncertainty when comparing to either CFD or measurements alone. Second, there are further theoretical aspects that have to be explored. For example, the LETKF in its current form assumes independence between the background and measurement errors; however, the measurements have been used for both setting up the numerical model and for assimilation, which could lead to a non-negligible correlation between the errors in the background state estimates and the measurements. Furthermore, the recent implementation of the LETKF is based on the assumption that the distribution of the observation errors is Gaussian, which might not be completely true [36]. Nevertheless, the difference to Gaussian noise is small as long as the SNR is sufficiently large [37–39]. The complex nature of the PC-MRI observation data emphasizes the need for data assimilation algorithms that are specifically adapted to hemodynamic problems. Lastly, the geometry was segmented based on the PC-MRI data and not on the highly resolved ToF data to reduce registration errors. This increases the uncertainty of the geometry used for the simulations due to the lower imaging resolution of the PC-MRI data, but reduces the uncertainty related to the registration procedure. We assume that the segmentation comprising the middle cerebral artery might have suffered from artifacts and does not match the true position very well, leading to a mismatch of boundary layers between the real geometry and the segmented counterpart. Nevertheless, the data assimilation step shifts the flow rate closer to the observations in comparison to the open loop (Fig. 3). A future data assimilation study would account for both sources of uncertainty (registration and resolution) simultaneously while keeping track of the true position of the vessel walls. A more sophisticated strategy for the segmentation of the vessel boundaries by the use of non-rigid registration between highly resolved ToF data and the PC-MRI flow field could account for this problem. Furthermore, the used observation operators can be optimized with respect to the PC-MRI data, by including the point-spread function of the acquisition sequence in the mapping function (e.g., sinc-function for idealized Cartesian sampling, temporal filtering) [16, 17, 36]. Finally, a sensitivity study to optimize the data assimilation parameters (including ensemble size and localization radius) is planned.

## Conclusion

In this study, we investigated the hemodynamics of a complete subject-specific circle of Willis using data assimilation. Fully 4D PC-MRI velocity measurements have been incorporated into numerical simulations using a local ensemble transform Kalman filter. The assimilation step has greatly reduced the uncertainty of intracranial state estimates in comparison to either CFD or measurement data alone. Although no ground truth is available here, the results can be used as a proof-of-concept for hemodynamic data assimilation in complex intracranial geometries. They demonstrate the benefit of combining multiple sources of data regarding key quantities, such as flow rates and wall shear stress when investigating intracranial hemodynamics. Contrary to variational-based techniques, the ensemble-based approach directly provides an estimate of the uncertainty. Future comparison between alternative techniques (e.g., [16–18]) needs to outline the strengths and weaknesses of different approaches.

## Abbreviations

CFD: Computational fluid dynamics
DA: Data assimilation
LETKF: Local Ensemble Transform Kalman Filter
PC-MRI: Phase-contrast magnetic resonance imaging
